# From Desperation to Sustainability: A Qualitative Exploration of Drivers and Barriers to Time-Restricted Eating in IBS Treatment

**DOI:** 10.3390/nu18060940

**Published:** 2026-03-17

**Authors:** Henrik Sverdrup, Asgeir Brevik, Maria Thompson Clausen, Marit Kolby, Marianne Molin

**Affiliations:** 1 Department of Nutrition, University of Oslo, Blindern, P.O. Box 1046, 0317 Oslo, Norway; henrik@baumann.no (H.S.); mthclausen@gmail.com (M.T.C.); 2 Department of Health and Exercise, Kristiania University of Applied Sciences, Sentrum, P.O. Box 1190, 0107 Oslo, Norway; marianne.molin@kristiania.no; 3 Institute of Nursing and Health Promotion, Oslo Metropolitan University, St. Olavs Plass, P.O. Box 4, 0130 Oslo, Norway; 4 Department of Health, Oslo New University College, Ullevålsveien 76, 0454 Oslo, Norway; marit.kolby@oslonh.no

**Keywords:** time-restricted eating, TRE, irritable bowel syndrome, IBS, drivers, barriers, feasibility, qualitative study

## Abstract

**Background/Objectives**: Irritable bowel syndrome (IBS) is a prevalent gastrointestinal disorder with implications for individual quality of life and society. Patients with IBS suffer a variety of symptoms but have few treatment options. The level of satisfaction with IBS treatment is low, stressing the need to expand the IBS treatment toolbox. The aim of this study is to describe drivers and barriers to the implementation of time-restricted eating (TRE) as a treatment alternative for patients with IBS. **Methods**: A convenience sample of 14 informants was drawn from a pool of 97 successful participants in an eight-week 16:8 TRE intervention. The informants partook in audio-recorded semi-structured in-depth interviews. Recordings were processed by a computer language model and interview transcripts were generated automatically. The transcripts were proofread, structured and analysed with a reflexive inductive thematic analysis approach. **Results**: The analysis generated six main themes consisting of 18 sub-themes in total. One main theme describes drivers of implementation concerning domains such as motivation, supporting factors, mentality, behaviour and determinants of sustainability. The results from this study are largely coherent with the findings from earlier feasibility studies conducted on other populations, but several key differences related to population characteristics emerged. **Conclusions**: Overall, the analysis suggests that TRE can be a feasible treatment option for IBS, but successful implementation is dependent on individual ability, external support and symptom relief.

## 1. Introduction

Irritable bowel syndrome (IBS) is a prevalent functional gastrointestinal disorder (FGID) characterized by symptoms such as abdominal pain, altered bowel habits, and bloating [1,2]. The condition frequently affects mental health and quality of life [3,4,5,6] and poses a significant global disease burden with a reported prevalence of up to 11% depending on diagnostic criteria and population [7,8,9]. Treatment typically focuses on symptom relief through medications, supplements or dietary interventions, but remains challenging as IBS is multifactorial in origin and symptoms and severity vary widely between individuals.

The low FODMAP (Fermentable Oligo- Di- and Monosaccharides and Polyols) diet has been shown to provide symptom relief for approximately 70% of patients with IBS [10], and is considered to be both safe and effective [11,12,13,14]. However, successful adherence to this diet often requires professional guidance, and is not effective for all patients [15,16]. In Norway, few patients with IBS report satisfaction with the treatment received [2], underscoring the need for additional, feasible treatment options.

Time-restricted eating (TRE) is an emerging dietary strategy that confines food intake to a set window each day, without specific restrictions on caloric content or food types. TRE typically extends the overnight fast to at least 14 h, and may be practiced as either early or late TRE depending on meal timing [17]. TRE has gained increasing scientific attention for its potential to positively affect metabolic markers, caloric control and weight reduction [18,19,20]. A variety of mechanisms are identified as possible causal factors for IBS symptoms, including intestinal inflammation, gut dysbiosis and intestinal dysmotility [20,21,22,23,24]. Intermittent fasting has shown potential to alter mechanisms associated with IBS, including bacterial dysbiosis, gut dysmotility, inflammation and altered gut microbiota [19,25,26,27,28,29,30], and has been suggested as a possible therapeutic treatment for IBS patients [31].

TRE feasibility has primarily been studied in the context of weight management in individuals with obesity, diabetes, and cardiometabolic risk, and the available literature is limited to this set of populations with similar characteristics [32,33,34,35,36,37,38,39,40,41,42]. It remains unclear whether the feasibility of TRE differs depending on the nature of the condition being treated. As patients with IBS face unique challenges, including gastrointestinal discomfort and mental health comorbidities, it is important to assess the feasibility of TRE in this specific group. A qualitative inquiry looking into patient experience and perception of the feasibility of TRE as a treatment option for people with IBS can serve as a basis for the informed planning and execution of future studies.

This study aims to explore and describe the key drivers and barriers to implementation of a 16:8 TRE pattern as a treatment for IBS. The study addresses the following research questions:

1. What are the drivers of successful implementation of TRE in patients with IBS, and how do they affect the feasibility of the treatment?
2. What are the barriers to the implementation of TRE in patients with IBS, and how do they affect the feasibility of the treatment?

## 2. Materials and Methods

### 2.1. Intervention

**Figure 1:**
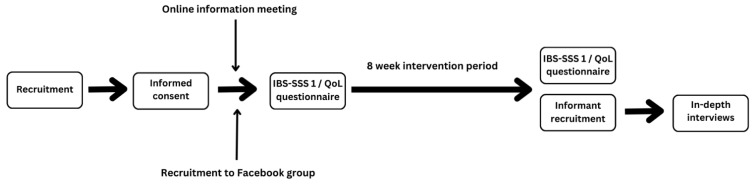
Overview of the study design and progression of the quantitative and qualitative components of the TRE pilot project.

This qualitative inquiry is embedded within a one-armed pre- and post-test pilot study [43] with the aim of exploring the efficacy and feasibility of TRE as a treatment for IBS (Figure 1). The participants in the pilot study completed an eight-week 16:8 TRE intervention. They were provided with explanatory material and close follow-up through a closed, private Facebook group.

### 2.2. Informant Recruitment

**Figure 2:**
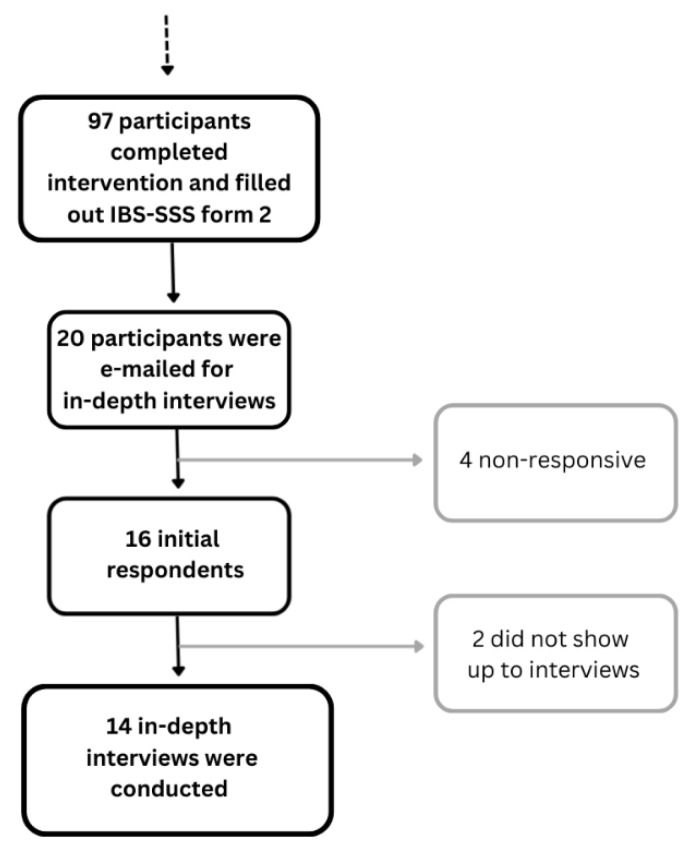
Recruitment flow chart describing the informant recruitment process in the qualitative part of the pilot project.

Informants were recruited from a purposive pool of participants who successfully completed the TRE intervention, and the informant recruitment goal was to acquire 12 to 16 informants. This interval was elected based on an assumption of high information power within the participant pool [44,45]. Of the 97 participants who completed the intervention, 20 were e-mailed and asked to be informants, including all the male participants who had agreed to be contacted for interviews. Beyond gender, the informants were selected based on age variability. Of the 20 recipients, 14 served as informants. We elected to only interview successful participants in order to have a sample of informants with a comparable basis for providing insight into the drivers and barriers to the intervention implementation, so as to avoid doubts concerning the information power of the sample. The process from the administration of the initial recruitment emails to the in-depth interviews is presented in Figure 2.

### 2.3. Data Collection

The data was collected through semi-structured in-depth interviews guided by five item interview guide initially tested in two pilot interviews that were later included in the dataset. The interview guide is provided in the Supplementary Materials (Table S1: Interview Guide). The interviews were conducted digitally and in solitaire via Zoom and recorded using the Diktafon app (https://nettskjema.no) with an additional offline backup recorder. Recordings were securely transferred to a digital workspace owned by the University of Oslo and operated and developed by the Tjenester for Sensitive Data (TSD) service group at the University of Oslo, IT-Department (USIT), (https://www.usit.uio.no). On average the interviews lasted 45 min, with no repeat sessions. The two first interviews were at first considered pilot tests but were later included in the data set as no changes were made to the interview guide. Consecutive notes were made during interviews in addition to the recordings. Transcripts were automatically generated using Whisper, a pretrained automatic speech recognition model provided by the University of Oslo. The transcripts were sequentially proofread and structured, resulting in 154 pages of data. The informants were informed of their right to access their transcripts, although none requested them.

### 2.4. Data Analysis

The data was analysed using an inductive reflexive thematic analysis approach, following Braun & Clarke [46]. This data-driven method was applied to identify patterns without relying on predefined frameworks, allowing the findings to emerge from the data through the lens of the analyst. The process of analysis followed Braun & Clarke’s six phases of thematic analysis: (1) data familiarization, (2) initial coding, (3) theme searching, (4) theme review, (5) theme definition and naming, and (6) report production. Table 1 illustrates the theme generation process:

### 2.5. Reflexivity

This data collection and analysis was based on the master’s thesis in clinical nutrition of a male 29-year-old Norwegian (H.S.). The researcher has, for a period of several years and ongoing, practiced an eating pattern reminiscent of TRE as a self-treatment for gastrointestinal complaints and weight control without having been diagnosed with IBS or any similar diagnosis. The researcher’s subjective experience with his gastrointestinal affliction and success in treatment with TRE must be considered as an influence on the researcher’s view on both the effectiveness and feasibility of TRE. The researcher had no prior experience conducting in-depth interviews or qualitative analysis. Apart from the intervention lectures, the Facebook group, and recruitment e-mails, the informants had no contact with the researcher or knowledge of the researcher’s personal motivation for doing the research.

### 2.6. Ethics Statement

The pilot project was approved by SIKT (project number: 942235) and the Regional Committee for Medical and Health Research Ethics (REK) (project number: 744378). The pilot was a collaboration between the University of Oslo, Oslo New University College, Oslo Metropolitan University, Kristiania University College of Applied Sciences and the Norwegian Gastrointestinal Association. The Consolidated Criteria for Reporting Qualitative Research (COREQ) checklist [47] and the Reflexive Thematic Analysis Reporting Guidelines (RTARG) [48] were employed in the production of this paper.

This manuscript is based on the first author’s MSc thesis deposited in DUO at the University of Oslo in 2025. The thesis is not a peer-reviewed publication. Relevant sections have been substantially rewritten, and figures and tables have been reused with proper attribution and permissions. We disclose this information for transparency.

## 3. Results

### 3.1. Informant Characteristics

The 14 informants were all Caucasian women from different areas of Norway, aged between 23 and 66 years. Two were in their twenties, two were in their thirties, six were in their forties, one was in their fifties, and three were in their sixties. The informant group consisted of students, workers and retirees, singles, people living with their parents, in collectives, with small children or teens, or with spouses only.

### 3.2. Themes

The analysis generated six main themes consisting of 18 sub-themes (Table 2). Each sub-theme represents a single driver or barrier to TRE implementation among patients with IBS, whereas the themes represent overarching domains of the feasibility of the intervention.

#### 3.2.1. Initial Sources of Motivation

The informants’ initial motivation to embark on and complete the TRE intervention stemmed from two key drivers: a deep sense of desperation due to the burden of living with IBS, and a desire to contribute to scientific research that could benefit others with the same condition. The sources of motivation were an important premise for the informants’ successful implementation.

For many, participation was fueled by a growing dissatisfaction with existing treatment options and a perceived lack of support from the healthcare system. Several informants described feeling dismissed or unsupported in clinical encounters, receiving what they interpreted as a resignation to live with the condition and accept the status quo. As Lauren shared, “You will just have to live with this, you’ll just have to try and figure out what you tolerate and what you don’t. It was really like, here you go, here’s your IBS. Go fix it. Or just live with it”. This disillusionment, combined with the high toll and limited success in implementing the low-FODMAP diet, led many to view TRE as a potential alternative worth trying. Several informants expressed a hope that TRE might end their dependence on the low-FODMAP diet, essentially swapping one burden for a different, and hopefully lighter one. Lily reflected, “Perhaps food is the key, not only FODMAP, because that was difficult. I don’t really think that was the key. Or maybe it partially was the key, but I haven’t managed to turn it”.

The experience with chronic pain, discomfort, and reduced quality of life caused by IBS created a sense of desperation from which informants sought change. As Lauren explained, “I’m at a point in life where I cannot take the stomach ache any longer, I’m so tired of it… So I just threw myself into it and thought, ‘It has to be tried, I mean, I have to do something now’. I’m so tired of being in pain that I will join just about anything.” This desperation did not exist in isolation but interacted with and enforced other implementation drivers, shaping how informants approached the intervention and reinforcing their determination to persist.

In parallel with this personal urgency, many informants were strongly motivated by the opportunity to contribute to scientific inquiry, and through their own efforts help other patients with IBS. Even those who were uncertain about the personal benefits of TRE expressed a desire to “do something meaningful”. Sarah stated, “I wanted to join because those who get diagnosed at a young age see it as a dark future, and I want there to be solid research into IBS. Life can actually still be good even though you have got IBS”.

For some, the idea that their participation might help others outweighed their desire for symptom relief. Ella reflected, “It feels quite useful to feel like I’m part of something greater, rather than just having this as a project for myself. Or you know, if I drop out, it’s kind of like… Then I’ll just go back to having stomach aches, so that’s not such a terrible loss. But I feel like being part of something bigger has kept me in line”. A sense of accountability to the research project and gratitude that someone was addressing IBS in a new scientific context emerged repeatedly. “I’m very glad that someone chose to research this topic”, said the same informant, “and so I’m thinking, if I can join in and contribute, then I think that’s very exciting”.

#### 3.2.2. Barriers

Implementing TRE presented multiple challenges for informants, which coalesced into the theme Barriers. These challenges manifested as psychological, physical, and social difficulties, stemming from the need to relinquish familiar and comfortable habits, endure bodily discomfort, and maintain an eating pattern out of sync with the rest of the world.

A central difficulty reported by informants was the sense of renunciation, described as the emotional and social cost of forgoing established comfortable routines and pleasures. Renouncing everyday pleasures was a significant source of decision fatigue that the informants had to deal with. Several informants described a feeling of loss, particularly during shared meals and evening rituals. Forgoing social eating, especially during family breakfasts or relaxed evenings, was a common theme. One informant remarked, “I very much missed taking part in family breakfasts on the weekends. I would just sit there with a dull cup of tea, and, well…” (Lauren). The act of abstaining in these settings was often described as isolating, with others not fully understanding or accepting the behaviour of the abstaining individual. As Grace described, “As a student living with others, it’s been more about the evenings, in not being able to… We’re watching a movie or whatever, and then, no, I can’t, I can’t join in on eating popcorn or whatever it is. And it hasn’t always, socially, been seen as acceptable around me”.

The emotional sacrifices were compounded by significant physical discomfort during the early stages of implementation. The informants reported symptoms such as physical and mental fatigue, poor concentration, and heightened hunger, particularly during the fasting periods. Sophie shared, “My body felt stressed, and I would have a cold sweat, and I didn’t manage to concentrate. But that has passed now. But when it was at its worst, it was very bothersome”. Striking a balance between meal timing and volume was also a source of distress, as individuals initially struggled to find a rhythm that supported both satiety and digestive comfort. One informant noted, “I got a lot hungrier in the following fasting period. It was like I just… I struggled more with hunger if I just ate two times a day. So, for me, it’s better to have three meals a day. And then, eight hours feels a bit short, I don’t manage to get hungry again in time” (Rachel).

TRE often clashes with the structure of the informants’ everyday lives. This friction with life, described as a misalignment between new personal routines and societal norms and rhythms, created ongoing logistical and emotional strain. Chloe highlighted the difficulty of balancing family responsibilities with meal timing: “It’s the difficulty of having three kids and a dog and a lot of activities in the afternoon, and so I experienced that I often thought it was quite difficult to find time for a proper meal before seven O’clock”. The success of TRE frequently depended on meticulous planning and lapses often led to suboptimal eating. As Grace admitted, “Eighty percent of the time there was an improvement, and the rest of the time it was simply bad planning. I sometimes ended up eating differently, and then of course I didn’t eat optimally that day. But in general, with good planning and routines, there was an improvement”.

Ultimately, while many informants adapted over time, social and lifestyle-related barriers persisted. The cumulative burden of isolation, disruption, and hunger made adherence challenging, particularly at the beginning of the intervention. As one informant summarized, “Everything involving food, being served, coziness with others, the social aspects of food… That is the worst part. Of course, it’s uncomfortable to be hungry. But that’s not the worst part of it. It’s actually the social aspect that is the worst part of it” (Rachel).

#### 3.2.3. Implementation Mentality

A shared feature among informants who successfully implemented TRE was the development of a specific implementation mentality. The informants acquired a cognitive and motivational stance that helped them overcome both early and lasting challenges. Overall, this mentality was marked by predetermination, perseverance, commitment, and an evolving awareness of bodily signals and physical experiences.

Many informants entered the intervention with a strong sense of predetermination, having already decided to follow through regardless of difficulty. The decision was tied to an awareness of the limited time frame of the intervention, and confidence in one’s own ability to push through. This proactive mindset helped them resist temptations and maintain consistency. As one informant noted, “I still think it’s about mentality, that when you are going to implement something like this, then you have to be determined and decide that you want to succeed with it” (Lily). Others viewed participation as a unique opportunity they didn’t want to squander, describing a clear, upfront decision that eliminated the need for continuous self-negotiation. Emily reflected, “It hasn’t been difficult. It’s been manageable for my part. But I was so focused on the project, so happy that I got to participate, that I decided that I was going to complete this”.

Despite the varying levels of difficulty, the informants emphasized perseverance as a critical factor in managing the initial phase. For many, this meant mustering willpower in the face of discomfort and habit changes. Rachel described this as a finite resource: “A lot of it has to do with willpower. It would be nice if fewer things relied on willpower. I think it gets depleted sometimes”. However, once symptom relief became apparent, the effort required diminished, and perseverance became less relevant. Jane explained, “It’s like, OK, now you have to be more present, you have to be stricter with yourself. But mostly in the beginning, because during the process, after I noticed how much good this did for me, it got very easy to do”.

Another significant driver of adherence was a strong sense of commitment, especially to the study. Many informants described their involvement in the project as a motivating force that provided structure and a sense of accountability. “… but being part of the project, that saved me during that difficult period. Then it was like, you are going to pull through with this, you can do eight weeks, and then after that you can live as you like. But at least pull through these eight weeks” said Lauren. Several informants reported that external accountability to researchers, family, or the idea of contributing to science was more motivating than personal health goals alone. Ella admitted, “… and that’s why I felt like it helped a lot, that I had kind of… This is for a project, I have to complete it, because I notice that now, it is also a bit like… When the consequences only affect me, it gets a bit more difficult. So that made it easier to pull through”.

Finally, several informants described a growing awareness of their bodily cues, particularly hunger. This increased awareness allowed them to distinguish between physical hunger and emotional or habitual urges, which in turn built resilience. Sophie noted, “But now, it’s very easy to feel that… I feel hunger. Compared to the times when I have trouble in my stomach, when it’s hard to understand if I’m hungry, or if I’m bloated, or if I’m full. But now I can to a greater extent recognize hunger. The feeling of fullness, now I’ve eaten too little, now I need food, now I’m just tired, now I need to relax”. Others learned to view hunger not as an emergency, but as a manageable, temporary state. As Chloe put it, “I experienced that the hunger that comes, it actually only lasts for about 20 min. That really acute feeling of being desperate for food. So, if you can get through that, then there is no issue with this at all… I think that an additional lesson from this is that you can actually tolerate being hungry”.

#### 3.2.4. Supporting Factors

Several elements surrounding the TRE intervention helped the informants to overcome barriers and increased its feasibility. Informants consistently highlighted the value of community, flexibility, and the paradoxical support found in structured constraints that reduced the burden of daily decision-making.

The sense of community, primarily fostered through the dedicated intervention Facebook group, was especially important in the early phase of the intervention when uncertainty and difficulty were at their peak. The online forum provided expert support and peer encouragement. Insights into how others struggled or succeeded helped normalize individual experiences and foster a feeling of belonging. Sarah noted, “When someone wrote something on the Facebook-page, for instance, then it was like, oh, you feel like that too. So, you’re not completely alone in that”. Others emphasized how the group made the project feel “real”, thereby strengthening their sense of commitment. As Chloe put it, “It’s seeing it, and not just knowing it kind of theoretically, that you are part of a group. Just seeing that can have… It’s the sum of everything that creates that kind of commitment”.

A key facilitator of long-term adherence was the allowed flexibility that the informants had in changing the placing of the eating window from day to day. Many informants found that they could adapt TRE to fit their changing routines, which reduced friction with social events and family life. Lauren described adjusting the window for a party, noting, “It went completely fine. I just adjusted the window the following day”. Post-intervention, several informants adopted a hybrid approach, adhering strictly to 16:8 during weekdays, and relaxing to 14:10 on weekends, demonstrating how flexibility contributed to sustainability.

Over time, the self-imposed rules that some initially perceived as restrictions became a supportive structure that helped the informants stay on the course they had committed to. The informants appreciated how the rules of the intervention helped them override old habits and simplify everyday decision-making. Jane explained, “It’s been a lot easier to make wise decisions… When I see that it works, it makes me want to make different decisions”. This structure became something to lean on rather than resist and provided the informants with a reassuring notion of not being left entirely to their own devices. “I feel like I often make up excuses to myself, which keeps me from completing things. I was very clear with myself in that this is a rule. This time I have to complete.” said Chloe, describing how the self-imposed rules helped her remain accountable and maintain adherence.

Finally, many described a seemingly surprising sense of freedom from choice. With TRE acting as a clear guideline, informants no longer had to constantly debate what or when to eat, which reduced decision fatigue and temptation. Kate reflected, “It was very nice. I didn’t have all those choices… That was just the way it was, and the way it’s supposed to be”. This “voluntary compulsion”, as some described it, created a psychological environment in which implementation became more automatic and less effortful. The sense of freedom from choice originated from a variety of drivers among the informants, such as the notion of accountability towards the project, the researchers or fellow participants, the predetermination with respect to following the rules of the intervention, having managed to self-impose the rules, and eventually the effects from the intervention itself.

#### 3.2.5. Implementation Behaviour

The informants demonstrated a range of active strategies to overcome barriers and successfully integrate the TRE intervention into their daily lives. Central to this process was a proactive approach to facilitating their environment and social context, alongside ongoing adaptation to the challenges encountered throughout the eight-week period.

Many informants described how openly communicating the nature and purpose of the intervention to family, friends, and colleagues helped reduce social friction and garnered crucial support. Being upfront allowed others to understand and accommodate their changed routines, which some informants credited as instrumental in maintaining adherence. For example, one informant shared how honesty at work fostered acceptance: “I told my boss about it. That I might become a bit cranky. But I told him to let me know and tell me to get it together. And it went really well” (Sarah). The informants found the support they managed to muster from their surroundings to be important to their implementation process: “I had a lot of understanding around me, both at work and at home. When people around you show understanding… That really helps. When people support you, when you’re involved in such things. It’s really great support” (Nina).

Beyond social facilitation, informants actively adapted their eating habits to better fit their lifestyles and reduce discomfort. Small but strategic adjustments, such as modifying meal size and timing, were pivotal. One informant described how she successfully managed a narrow eating window around her children’s activities: “The kids have their activities at five, and they last to half past six. And so, I couldn’t get home to eat in time before the eating window closed. But I had eaten a proper dinner portion before that, and I thought; “let’s see how this goes”. And it went so well” (Sarah).

Dietary composition was also adjusted to manage hunger within the eating window, typically by increasing familiar satiating foods rather than introducing new ones. Informants emphasized the importance of patience and experimentation during the initial adaptation phase, highlighting that perseverance and allowing oneself time to learn from trial and error were key to finding individualized, sustainable routines. As one informant advised: “It can take time to figure out what works. I mean, I tried both early and late windows and discovered that a late window made implementation much easier for me. You’ve got to spend some time to try and fail a little, actually, before you give up. You’ve got to find your own way of doing it” (Anna).

#### 3.2.6. Sustainability

The informants’ experiences revealed sustainability as an ideal end destination for TRE implementation. The sustainability state is defined by a shift in the balance between the perceived costs and benefits of TRE, improved energy management, and formation of new habits.

Throughout the intervention, many informants underwent a transitional phase marked by physical discomfort, routine changes, and effortful self-control before symptom relief and ease of adherence emerged. One informant described this early struggle: “Week one was absolutely horrible. There was a constant feeling of hunger, I was completely focused on when I could eat” (Sarah). This difficult period typically lasted between three and six weeks, after which a notable shift occurred—symptom improvements coincided with reduced effort and increased motivation. This positive feedback loop allowed informants to move beyond reliance on sheer willpower: “I think it took about two or three weeks before it got easier, but the difficulty also disappeared along with when I noticed the symptom improvement” (Ella). “During the project, when I could feel that this was good for me, it got even easier to avoid those bad decisions” (Jane). The eventual benefit outweighed the costs, making continuation an obvious choice for the informants: “After the eight weeks, I fell of the wagon, and I bitterly registered what that did. And so now I’m back at it again. I am. Because I have a much better everyday life, and a much better life in general. So… Yeah… The fact that it actually took that little” (Kate).

Parallel to the cost–benefit balance shift, informants reported an increase in general energy, distinct from symptom relief, that helped counteract the decision fatigue caused by renunciation and sustained effort. This energy boost improved their ability to cope with stress and decision fatigue, and maintain TRE: “But you know, it’s the strangest thing. My energy is at a whole other level than it’s been before. I can handle stress a lot better” (Sarah).

Lastly, habit formation was crucial to reaching the state of sustainability. The structured eight-week intervention provided the consistency needed to embed TRE into a natural routine. While the time to establish a habit varied from less than two weeks to six or seven weeks, all the informants agreed that the duration of the intervention was sufficient: “Being in the project made it possible for me to get into the routine. Because it lasted that long. If it had only been for three weeks, I might have gone back to the usual. But since it lasted for two months, I managed to get into it, and so now I’m still doing it” (Lauren).

Several informants described the completion of habit establishment as when the new behaviour felt like the most natural course of action, involved the least friction, and aligned with what they now identified as. Establishing TRE as a habit seemed to involve a redefinition of oneself, becoming a new person who acts in a specific way. In this sense, the informants did not only eat time restricted but became time-restricted eaters. Informant number 6 describes it like this: “… but I don’t eat now. It’s kind of like… It’s not about what to eat, or thinking no, you shouldn’t. It’s about not having the desire to have that slice of bread… Because it’s just… No. I don’t eat now”. The personal redefinition, closely tied to symptom relief solidified TRE as the preferred lifestyle, making any deviation feel counterintuitive: “I’m going to continue with this. It’s as if the body… it doesn’t want to do anything other than this” (Sophie).

#### 3.2.7. The Process of Change

**Figure 3:**
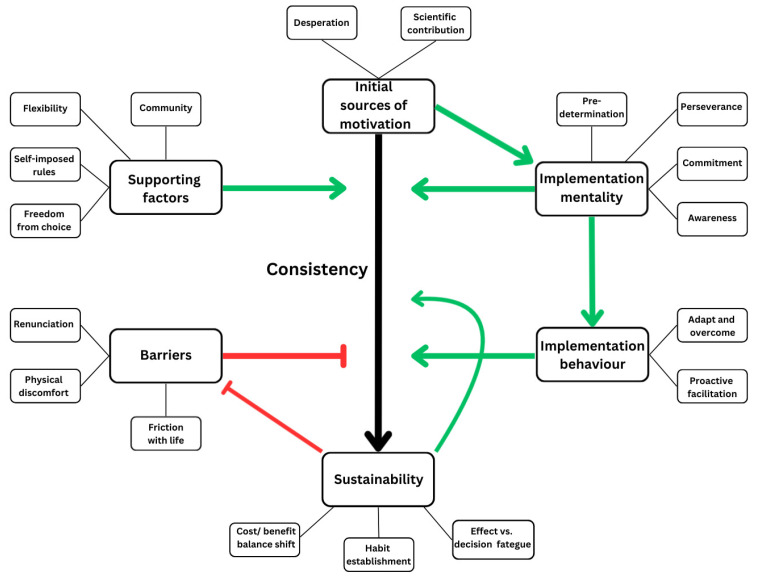
Thematic map illustrating drivers and barriers to TRE implementation among individuals with IBS. The figure visualizes the relationships between the six main themes and their 18 sub-themes. Arrows indicate reinforcing (green) and inhibiting (red) relationships that influence consistency in adherence and the transition from initial implementation to sustainability.

The search for drivers and barriers to implementation resulted in a group of themes that, in relation to each other, acted as determinants for a process of transition. In sum, the collection of experiences describes a transition from a state of gradual depletion of mental resources to a positive spiral of feasibility. Recurring across themes, consistency in adherence stood out as key to entering the positive spiral. The themes represent key factors influencing feasibility but relate to consistency in different ways. The relationships between themes and their role in the transition process is described in Figure 3.

The sources of motivation tied to IBS act as drivers of initial adherence. Additionally, they serve as a foundation for acquiring the necessary implementation mentality, which again facilitates implementation behaviour. Accompanied by supporting factors, the four main themes are important drivers of consistency, and consequently the feasibility of TRE. The barriers on the other hand, are antagonists to consistency, and must be overcome for long enough to reach sustainability. The necessary duration of consistency to reach sustainability depends on the informants’ susceptibility to being affected by the barriers and varied substantially between the informants. In this intervention, reaching a state of sustainability took at least one week, typically four to five weeks, but also as much as seven to eight weeks.

The positive spiral of feasibility is the point at which the intervention became sustainable. Through consistent adherence, the informants achieved symptom relief, shifting the balance between cost and benefit. Accompanying symptom relief was a general increase in energy that further increased the informants’ ability to adhere to the intervention. Following sufficient time and effect, the informants started forming habits, which reduced the cost of intervention, making adherence increasingly easier. With the increased resilience from the effects described in energy vs. decision fatigue, the increased motivation resulting from the cost–benefit balance shift and the increasingly easier adherence from establishing habits, the barriers to implementation eventually diminished. At this point, the sources of motivation, supporting factors and implementation mentality have become redundant, implementation behaviour largely has become habitual behaviour and the state of sustainability acts as its own driver of further consistency.

## 4. Discussion

The results showed that informants were initially driven by desperation due to chronic IBS pain and discomfort and a desire to contribute to research benefiting others with IBS. Barriers included psychological, physical, and social difficulties. The informants employed a proactive implementation mentality and adaptability to overcome these barriers. Supporting factors, such as community and structured constraints, aided adherence, while strategies, such as social facilitation and dietary adjustments, helped integrate TRE into daily life. The informants’ experiences suggest that TRE sustainability is achieved through a beneficial shift in the balance between costs and benefits, improved energy management, and habit formation, which leads to lasting changes in behaviour.

### 4.1. In Light of Previous Research

The barriers described in this paper share extensive overlap with descriptions in earlier reports, suggesting little discrepancy in the domains of difficulty in implementation across different populations. Furthermore, both previously established and novel drivers have appeared, indicating both similarities and important differences between conditions in terms of how successful TRE implementation can be achieved. The motivational drivers identified in this analysis (desperation and scientific contribution) have not been described in TRE feasibility studies involving obesity, diabetes, or cardiometabolic risk populations [32,33,34,35,36,37,38,39,40,41,42]. The urgency expressed in the sub-theme desperation aligns with the descriptions of IBS patient experiences reported by El-Salhy and colleagues [5], but does not appear in literature on TRE feasibility, suggesting a key distinction in feasibility based on patient characteristics. Similarly, scientific contribution, where participants expressed a desire to help others in their group, contrasts with findings from populations with obesity showing negative ingroup bias [49].

The barriers described in this analysis mirror those in previous TRE studies. Most pronounced are the challenges with social eating during events like holidays, parties and everyday meals [32,33,35,37,38,39,42]. As with renunciation and friction with life, earlier research also suggests that physical presence alone seems insufficient to fully experience the social benefits of shared eating. Descriptions similar to physical discomfort were frequently reported in other studies, including hunger, low energy, nausea, and difficulty adjusting to meal timing [36,38,39,40], echoing the findings from our study.

The theme of implementation mentality matched drivers described in previous research. Predetermination aligns with earlier descriptions of a beneficial preparatory mindset [34]. Perseverance has been noted as a maintenance factor [33], whereas we found its relevance to patients with IBS to be short-term, in the initial stage of implementation. Commitment also reflected prior findings; although accountability was described as demotivating and deterring in diabetics [33], it was a positive force in patients with IBS. Supportive factors such as community parallel effects of peer and professional support found in diabetes and obesity studies [34,42], as well as in cancer patients [50].

The importance of flexibility is well-established in the literature, as rigid eating windows and limitations in allowed beverages have been shown to correlate with reduced adherence while increased flexibility does the opposite [33,35,38,41,42]. Self-imposed rules and freedom from choice, while occasionally reported and described [39], were found to be uniquely positive in this study. The interplay of perceived self-imposed rules, commitment, and mindset forming freedom from choice has not been described elsewhere.

While the theme of implementation behavior is absent from previous literature, its components, such as the importance of social support [32,33,37,38,42] and adaptive strategies [32,37,38,39] are commonly noted. Proactive facilitation shifts the focus to how support is obtained rather than the impact of its presence. Adapt and overcome also mirrors prior descriptions of overcoming misalignments with daily routines [32,37,38,39]. Sustainability, proposed here as a self-reinforcing state representing an ideal end destination in the implementation process, is a novel concept. While determinants such as cost/benefit balance shift and generation of habits are unreported, secondary benefits (e.g., improved sleep, energy) have been described [35,36,38], supporting the potential for lasting implementation seen in effect vs. decision fatigue.

### 4.2. Methodical Considerations

Sample size determination in qualitative studies is usually guided by data saturation, concerning the number of informants needed for information redundancy [44,51]. However, the relevance of data saturation depends on the analysis method and is not universal for determining sample sizes [45]. In reflexive thematic analysis, the process of analysis has no theoretical endpoint and relies on the researcher’s interpretation. Without pre-existing frameworks such as those used in grounded theory, codes and themes are generated inductively by the researcher [52], and the analyst decides when the research question is considered answered. The analytic strategy of this study does not align with data saturation, as no framework exists to saturate [44]. With access to purposive informants, the concept of information power guided the sample size determination [44,45]. Unlike data saturation, information power does not specify informant numbers but explains why any number can suffice based on sample purposiveness. It focuses on whether sufficient information exists for a meaningful analysis rather than reaching saturation [44,45]. Although it was not used for sample size determination here, the interval of 12 to 16 informants aligns with what data saturation would suggest [53].

Data familiarization is a necessary first step to analysis and can be achieved through different approaches. Manual transcription is considered necessary by some [54,55], as it allows researchers to study information communicated beyond words. A silence in conversation carries meaning, unlike the gaps in automated transcripts. Manual transcription engages researchers through prioritization, suggesting it is an act of interpretation. In familiarizing with this dataset, some transcript portions were omitted as non-functional, although this differs from the interpretive nature of manual transcription. Furthermore, in inductive thematic analysis, coding differs from theory-based methods. Theory-driven approaches use preconceptions to generate codes, whereas reflexive data-driven methods allow researchers to subjectively determine interesting elements and coding approaches. Codes emerge from the researcher’s interpretation of the data, making the researcher integral to the analysis [46].

### 4.3. Limitations

The study questions were answered based on experiences from Caucasian women only, limiting the applicability of the findings. During informant recruitment, participants who experienced positive effects from TRE were unavoidably over-represented. Although their experiences of difficulty and symptom relief varied, all informants reported meaningful improvement and continued practicing a modified TRE after the intervention. While the informants were able to describe implementation challenges, they were generally considered minor or temporary. The feasibility assessment would likely differ if less successful participants were also interviewed, particularly with respect to the relationship between drivers and barriers over time, and the concept of sustainability as an achievable implementation destination.

Unlike cardiometabolic patients who experience gradual benefits such as weight loss and lower disease risk, the informants in this study reported the symptom relief from TRE to be sudden when it first appeared. Due to this relatively rapid shift, the related drivers such as cost/benefit balance shift and effect vs. decision fatigue are specific to IBS patients. Implementation drivers such as scientific contribution, predetermination, commitment, forced structure and community emerged from study participation and intervention structure. Although these drivers can be replicated in future TRE interventions, current insights are insufficient for application in general IBS treatment settings and require further targeted research.

The data collection in this study relied on social interactions between the researcher and the informants, making it susceptible to participant bias. Social desirability bias occurs when informants perceive what is socially acceptable in interviews [56]. Given the intimate nature of IBS symptoms, this bias could limit informants’ descriptions. Additionally, informants might describe TRE as more feasible than experienced, knowing that this would please researchers. During pre-intervention presentations, the informants received implementation recommendations and challenge warnings, potentially causing observer-expectancy bias that could alter behavior based on perceived researcher expectations [57]. Lastly, the Hawthorne effect can affect interviews as participants may change their behavior simply due to being observed [58].

Even though the participants were instructed not to make additional, intentional dietary changes, studies have shown that TRE can lower an ad libitum energy intake by 7 to 22% [59], possibly inducing metabolic effects like weight loss. For informants with a desire to lose weight, this might have influenced their attitude towards TRE. Additionally, given the likely change in overall diet composition due to changes in meal patterns, such as skipping breakfast, we cannot rule out changes in diet composition that can alter IBS symptoms independent of timing effects.

### 4.4. Focus Points for Future TRE Implementation in IBS Patients

If we were to redo this study based on what we have learned, there are several focus points we would have included in the planning and execution of a new intervention protocol. These focus points are suggestions for what researchers should consider to successfully implement a TRE intervention in an IBS population.

1. Allow a sufficient intervention period. Many informants reported that TRE only became sustainable after 4–6 weeks, once physical adaptation and symptom relief occurred. Future studies should ensure the intervention is long enough for this transition phase to unfold.
2. Ensure mental readiness before enrollment. Require participants to engage in pre-intervention reflection or orientation sessions that emphasize realistic expectations, potential discomforts, and personal commitment. Encourage participants to visualize common barriers and decide in advance how they will maintain adherence.
3. Promote a single, sustained commitment. Introduce the idea of making one firm decision to adhere to TRE throughout the study in order to reduce decision fatigue. Reinforce this by asking participants to communicate their commitment to family and peers for accountability and practical support.
4. Normalize temporary challenges. Explicitly inform participants that physical, social, and psychological challenges are common but typically transient. Providing real testimonials or case examples can improve perseverance during the adaptation period.
5. Facilitate peer connection and researcher presence. Create structured communities where participants can share progress and experiences. Active researcher engagement through these channels helps maintain motivation and a sense of belonging, shared purpose and accountability.
6. Encourage strategic flexibility. Allow flexibility in meal timing within the 16:8 framework to reduce social strain and improve adherence. Provide practical tools and examples for adjusting eating windows without compromising intervention integrity.
7. Support proactive adaptation and problem-solving. Guide participants to anticipate and manage barriers through concrete actions: meal prepping, communicating dietary routines to others, planning for social events, and avoiding high-risk situations for nonadherence. Emphasize that adaptation is gradual and iterative.

## 5. Conclusions

This study demonstrates that TRE can be a viable treatment option for IBS patients as long as the positive effects on symptoms, health and energy outweigh the social, mental and physical costs. The results indicate that TRE can be sustained long enough for habit establishment and serve as a long-term sustainable treatment option for IBS patients.

## Figures and Tables

**Table 1:** Descriptive example of the coding and theme generation process of the analysis, where key information elements from the transcripts were collated and condensed into codes, sub-themes and main themes.

Main Theme	Sub-Theme	Code	Key Information Element
Barriers	Renunciation	Not being able to partake	“I very much missed taking part in family breakfasts in the weekends. I would just sit there with a dull cup of tea.”

**Table 2:** Overview of the six main themes and their composition of the 18 sub-themes making up the described determinants of TRE feasibility.

Main Themes	Sub-Themes
Initial sources of motivation	Desperation
	Scientific contribution
Barriers	Renunciation
	Physical discomfort
	Friction with life
Implementation mentality	Predetermination
	Perseverance
	Commitment
	Awareness
Supporting factors	Community
	Flexibility
	Self-imposed rules
	Freedom from choice
Implementation behaviour	Proactive facilitation
	Adapt and overcome
Sustainability	Cost/benefit balance shift
	Effect VS. decision fatigue
	Habit establishment

## Data Availability

The dataset presented in this article is not available due to the personal nature of the information it contains, and legal limitations concerning the distribution of personal information.

## Supplementary Materials

The following supporting information can be downloaded at: https://www.mdpi.com/article/10.3390/nu18060940/s1, Table S1: Interview Guide.

## Abbreviations

The following abbreviations are used in this manuscript:

TRE	Time-restricted eating
IBS	Irritable Bowel Syndrome
