# Supplementary material for: From Desperation to Sustainability: A Qualitative Exploration of Drivers and Barriers to Time-Restricted Eating in IBS Treatment
Source: Nutrients. 2026 Mar 17;18(6):940. doi: 10.3390/nu18060940 (PMC13029116; doi:10.3390/nu18060940)
Supplement: Supplementary file 1 [file nutrients-18-00940-s001.zip › nutrients-4178847-supplementary.pdf]

Table S1. Interview Guide.

**Main Questions**	**Possible Follow-up Questions**
**Opening Questions**	
a. Can you tell me why you chose to participate in this study?	
b. Approximately how long have you had an IBS diagnosis?	
c. Were you familiar with “time-restricted eating” before participating?	
**Theme 1: Feasibility and perceived outcomes of the intervention**	
a. How did you experience being a participant in the project?	How feasible did you find the intervention to be? Challenging? If so – what did you find challenging?
b. Tell me about your experiences with the intervention – did you feel that TRE either improved or worsened your IBS symptoms – and if so, which symptoms?	Probe. Please elaborate.
c. Did you experience that TRE helped with/affected anything beyond IBS symptoms?	Probe. Please elaborate.
d. Can you tell me how your experience of practicing time-restricted eating developed over the eight weeks? Did it become easier or harder over time?	When did you start noticing changes? Why do you think it developed that way?
e. What is your perception of what you had to give up in order to complete the intervention? Did you miss anything in particular?	Challenges related to, for example, skipping breakfast, etc.
**Theme 2: Factors that facilitated participation**	
a. How would you describe your motivation for participating?	Is perceived benefit central to feasibility?
b. What was important for you in order to be able to complete the intervention?	For example: support from family, friends, workplace (social support), Facebook group
c. If someone you know were to start with TRE, what advice would you give them?	What should they do / what should they avoid?
**Theme 3: Barriers to participation**	
a. What challenges did you expect to face with TRE before starting?	Probe: e.g. meal patterns, hunger, social situations, other family members’ routines, etc.

b. What did you actually find challenging about completing the intervention?	Probe: e.g. meal patterns, hunger, social situations, other family members' routines, etc.<br><br>Menstrual cycle?
c. How did you handle the challenges you encountered?	What did they require of you?
d. Did you experience any challenges that make you uncertain whether you could maintain TRE indefinitely / over a significantly longer period?	
**Closing Question**	
Is there anything you would like to add that I haven't asked about?	
